# Multimodal laminar characterization of visual areas along the cortical hierarchy

**DOI:** 10.1162/IMAG.a.1279

**Published:** 2026-07-14

**Authors:** Alessandra Pizzuti, Pierre-Louis Bazin, Dimo Ivanov, Sebastian Dresbach, Judith C. Peters, Rainer Goebel, Omer Faruk Gulban

**Affiliations:** Department of Cognitive Neuroscience, Faculty of Psychology and Neuroscience, Maastricht University, Maastricht, Netherlands; Brain Innovation B.V., Maastricht, Netherlands; Full brain picture Analytics, Leiden, Netherlands; Department of Neurophysics, Max Planck Institute for Human Cognitive and Brain Sciences, Leipzig, Germany

**Keywords:** cortical layers, 7T, microscopy, qMRI, R_2_^*^, vision

## Abstract

Understanding how brain structure gives rise to function remains a central challenge in neuroscience. Post-mortem histology provides unparalleled microstructural insight into cytoarchitecture, myeloarchitecture, and cell-type distributions, yet lacks information on functional coupling. Conversely, in-vivo MRI can reveal functional dynamics but with limited microstructural specificity. Bridging these modalities is, therefore, essential for advancing our understanding of cortical organization. Here, we investigate laminar organization across the human visual hierarchy by integrating post-mortem and in-vivo imaging. Specifically, we combined post-mortem histology and quantitative MRI (qMRI) from the Alkemade and colleagues’ dataset (Alkemade et al., 2022) with in-vivo ultra-high resolution q $T_{2}^{*}$ MRI (0.35 mm isotropic) and resting-state layer-fMRI (0.8 mm isotropic), focusing on areas V1, V2, V3, and hMT+. Among post-mortem measures, parvalbumin (PV) interneuron distributions across cortical layers best discriminated visual areas, outperforming cell body density (Nissl) and fiber density (Bielschowsky). Furthermore, comparing laminar $R_{2}^{*}$ profiles across modalities revealed systematic differences between post-mortem and in-vivo MRI, attributable to the absence of vascular contributions in post-mortem data. Extending these laminar analyses to resting-state fMRI acquisition (here only the temporal mean is used for the laminar profiles) represents a first step toward linking structural and functional profiles across layers. Finally, we make our analysis framework publicly available to enable broader exploration of laminar organization across cortical systems using the Alkemade et al. (2022) dataset. This integrative approach sets the stage for future frameworks that unite microstructural and functional data, advancing the development of next-generation models of cortical computation.

## 1 Introduction

Understanding the functionality of any physical component fundamentally requires exploring the relationship between its structure and function. This principle is a cornerstone of neuroscience, where one of the field’s enduring goals is to elucidate how brain structure underpins its myriad of functions. Post-mortem histological studies using microscopic techniques with micrometer-scale resolution have revealed that cortical architecture is highly heterogeneous. Analyses of cytoarchitecture (laminar distribution of cell bodies) and myeloarchitecture (laminar distribution of myelinated fibers) provided the basis for cortical parcellation (Amunts et al., 2013; Brodmann, 1909; Nieuwenhuys, 2013; Vogt, 1906; Zilles & Amunts, 2010). Complementing these approaches, post-mortem immunohistochemistry identifies the distribution of specific cell types across layers, such as excitatory and inhibitory neurons. While not used directly for parcellation, these data are critical for understanding brain function, as the balance between excitation and inhibition maintains cortical homeostasis, and its disruption has been linked to neurological disorders (Ferguson & Gao, 2018; Fotiadis et al., 2023; Markram et al., 2004; Torres-Gomez et al., 2020; Tremblay et al., 2016). Together, these post-mortem methods provide unparalleled cellular and microstructural detail but cannot capture functional coupling of microcircuits, as functional measurements cannot be routinely conducted on the same tissue (Turner, 2013b) (despite few exceptions Boon et al., 2019; Jonkman et al., 2019).

Magnetic resonance imaging (MRI) provides a complementary, non-invasive approach for studying both structure and function in the living human brain (Bandettini et al., 1992; Ogawa et al., 1992). Advancements in ultra-high magnetic fields have enabled submillimeter spatial resolution, allowing in-vivo imaging of the cortical landscape at the mesoscopic scale (<1 mm) (Koopmans & Yacoub, 2019; Shmuel et al., 2007; Stirnberg et al., 2024; Ugurbil et al., 2003). Structural scans can now reach 0.35 mm isotropic resolution (Bollmann et al., 2022; Gulban et al., 2022, 2026; Lüsebrink et al., 2021; Turner, 2013a), while layer-fMRI studies achieve functional imaging at 0.8 mm isotropic resolution, approaching the scale of cortical layers (De Martino et al., 2013, 2015, 2018; Dijk et al., 2021; Dresbach et al., 2024; Fracasso et al., 2016, 2021; Huber et al., 2017; Muckli et al., 2015; Petro & Muckli, 2017; Pizzuti et al., 2023, 2025; Viessmann & Polimeni, 2021; Zimmermann et al., 2011). Despite these advances, in-vivo methods provide limited microstructural detail compared with post-mortem histology, creating the complementary gap. A potential solution is the integration of multiple modalities. Previous multimodal approaches have demonstrated the value of combining post-mortem microstructural data with in-vivo functional and topographical imaging, enabling, for instance, the development of multimodal cortical parcellations (Glasser et al., 2016). Similarly, quantitative MRI (qMRI) techniques combined with modeling provide microstructural information to complement functional imaging (Dinse et al., 2015; Trampel et al., 2019; Weiskopf et al., 2021). Among qMRI measures, $R_{2}^{*}$ ($R_{2}^{*}$ = 1/$T_{2}^{*}$) relaxation rates are especially informative, indexing both microstructural properties such as iron and myelin (Deistung et al., 2013; Fukunaga et al., 2010; Marques et al., 2017; Weiskopf et al., 2021) and vascular architecture (Gulban et al., 2022, 2026). This is directly relevant to fMRI, since the blood oxygen level-dependent (BOLD) signal is predominantly $R_{2}^{*}$-weighted, linking microstructural and vascular tissue properties to functional signals (Markuerkiaga et al., 2021; Marquardt et al., 2020; Uludag et al., 2009). Here, we present a multimodal study that integrates post-mortem histological and qMRI data with in-vivo high-resolution quantitative MRI and resting-state layer-fMRI to investigate the laminar organization of the human visual hierarchy. We focused on four visual areas spanning hierarchical levels: V1, V2, V3, and hMT+. For the post-mortem component, we used a recently published open-access dataset comprising two human brains (Alkemade et al., 2022), which uniquely includes multiple histological contrasts (cytoarchitecture, myeloarchitecture, and immunochemistry) and qMRI measures from the same tissue (7 Tesla). In parallel, we collected high-resolution q $T_{2}^{*}$ MRI (0.35 mm isotropic) and resting-state layer-fMRI (0.8 mm isotropic) from a healthy participant at 7 Tesla. Using these datasets, our laminar analyses addressed three main questions: (1) in post-mortem data, which microscopy contrast laminar profiles best discriminates the visual areas of interest; (2) how laminar variations of $R_{2}^{*}$ compare between post-mortem tissue and in-vivo brain, reflecting tissue properties alone versus tissue plus vasculature; and (3) whether laminar variation of anatomical $R_{2}^{*}$ is reflected, to some extent, in resting-state fMRI signals. Answering these questions by using newly available dataset (Alkemade et al., 2022) required the development of novel analysis tools, which we implemented and made our pipeline openly available. Our multimodal approach provides a unified framework for linking microstructural and functional information across cortical layers, offering new opportunities to investigate structure–function coupling in the human visual system and beyond.

## 2 Materials and Methods

**Fig. 1.**
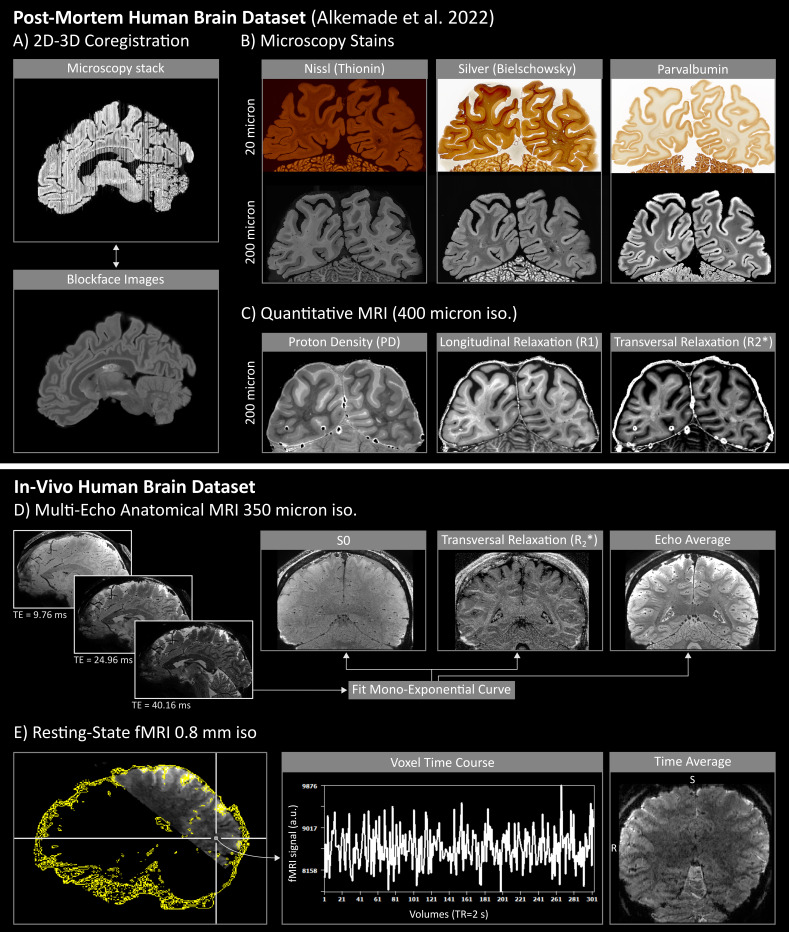
Data overview. (A–C) Post-mortem human brain (Alkemade et al., 2022) consisting of three microscopy stains: Nissl (Thionin) as neural density, Silver (Bielschowsky) as fiber density, and parvalbumin as interneurons density correlate (B) and three qMRI contrasts: proton density (PD), longitudinal relaxation (*R*_1_), and transversal relaxation ($R_{2}^{*}$) (C). Original stains were collected at 20 micrometers, while qMRI were collected at 400 micrometers. Both modalities were co-registered in the same brain space at 150 x 150 x 200 micrometer resolution through 2D–3D registration techniques with respect to the blockface images (A). In-vivo human brain dataset consisting of four runs of whole brain multi-echo multi-shot anatomical MRI at 350 micrometer isotropic resolution (D) and resting-state fMRI data at 0.8 mm isotropic with occipital coverage (E). Three echoes were collected and used to fit a mono-exponential curve and compute S0 and $R_{2}^{*}$ (D). rs-fMRI brain coverage and a voxel time course are shown in panel (E). Co-registration between anatomical and fMRI data was performed between the average echoes and average time course images.

**Fig. 2.**
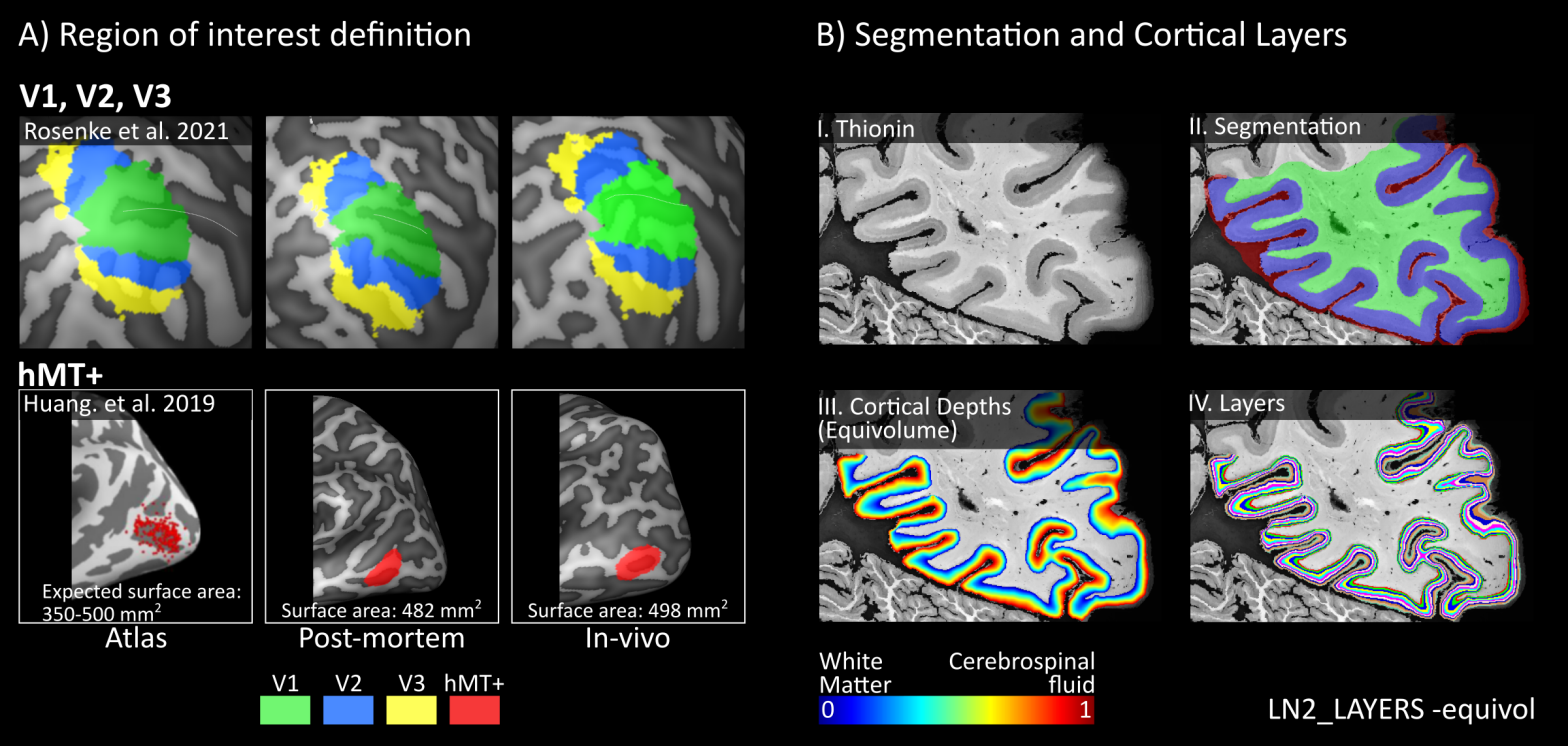
Overview of the two methodological steps. Panel (A) schematically illustrates the procedure used to define the regions of interest. A cortex-based alignment to the functional visual atlas (visfatlas, Rosenke et al., 2021 was used to define V1, V2, V3, while a macroanatomical procedure guided the definition of hMT+ to match a probabilistic atlas (Huang et al., 2019). Inspect Supplementary Figure S2 for an extended overview of our ROI definition. Panel (B) illustrates an example of input (i) for the computation of the geometric cortical layers using the program LN2_LAYERS with the -equivol option. As output, normalized equivolume cortical depth measure (iii) is discretized in 11 equivolume layers (iv). Note that 11 layers are chosen here as an example for visualizing discrete layers; for the main results, the entire cortical depth (iii) is used to compute layer profiles as 2D histograms.

Our study is based on two distinct human brain samples: (1) a post-mortem brain, from the publicly available 3D whole-brain microscopy, and 7T quantitative MRI (qMRI) data, from Alkemade et al. (2022), and (2) an in-vivo brain from a healthy human participant, for which we collected 7 Tesla whole-brain qMRI ($T_{2}^{*}$) at 0.35 mm isotropic resolution and resting-state fMRI at 0.8 mm isotropic resolution. An overview of the data is provided in Figure 1. While both datasets underwent the same conceptual analysis steps, the implementation was tailored to optimally accommodate the characteristics of each dataset. Our new pipeline’s main processing steps for laminar analysis can be summarized as follows: (1) cortical mapping involving region-of-interest (ROI) definition and tissue segmentation, (2) data preprocessing tied to extract laminar information, and (3) geometrical cortical layer delineation. We focused our laminar analysis on selected visual areas spanning progressive stages of visual hierarchy, such as V1, V2, V3, and hMT+ (Fig. 2). Early visual areas (V1, V2, V3) were defined based on the probabilistic visual functional atlas (visfatlas) (Rosenke et al., 2021), while hMT+ was defined based on the atlas published by Huang et al. (2019). Our analysis pipeline is publicly available on Github (https://github.com/27-apizzuti/multimodal_layers) and provides a new framework for analyzing the dataset of Alkemade et al. (2022), facilitating future studies on laminar cortical organization targeting ROI beyond visual areas.

### 2.1 Data acquisition

#### 2.1.1 Post-mortem dataset

The post-mortem dataset from Alkemade et al. (2022) is the first publicly available dataset containing a 3D whole-brain map of multiple microscopy contrasts and 7T qMRI from two human specimens. One specimen from this dataset (ID: 122017, female, 59 years old) was selected for the highest signal and reduced artifacts in the occipital part of the brain. Coronal slices with in-plane resolution of 0.021 mm and 0.20 mm across slices were stained for five microscopy contrasts in an interleaved fashion: two histology stains—Nissl (Thionin, glial, and neuronal cell body density) and silver stain (Bielschowsky, fiber density)—and three immunochemistry stains—calbindin, calretinin, and parvalbumin (interneurons density). Bielschowsky silver staining highlights axons and dendrites via metallic silver deposition and is commonly used to visualize nerve fibers and neurodegenerative pathology such as neurofibrillary tangles and amyloid plaques (Laule & Moore, 2018; Moore et al., 2000; Uchihara, 2007). Importantly, high fiber density does not necessarily imply high myelin content (and vice versa), as individual axons can vary substantially in their degree of myelination (Uchihara, 2007). Note that calbindin and calretinin were only available at the medial part (central) of the brain, not covering the visual areas of interest. Example slices at original resolution are shown in Figure 1B. Before sectioning, 7T whole-brain qMRI was acquired at 0.4 mm isotropic resolution, yielding proton density, R1, and $R_{2}^{*}$ maps. We focused on $R_{2}^{*}$ maps, as they represent the closest proxy to the $T_{2}^{*}$-weighted BOLD contrast primarily used in functional imaging (Fig. 1C). For this study, we used the preprocessed dataset shared by Alkemade et al. (2022), in which 2D microscopy images were aligned and reconstructed into a 3D multi-contrast staining dataset co-registered with the qMRI data at 0.2 mm isotropic resolution (as indicated in Table 1, step 2 by original authors). During dataset preparation, blockface images acquired during sectioning were reconstructed into a 3D volume at 0.15 × 0.15 × 0.2 mm resolution and served as the reference space for co-registration. This preprocessing allows a direct link between microscopic features and MRI slices, enabling the study of the microscopic underpinning of MRI contrasts. For a detailed description of the post-mortem data collection and reconstruction, refer to the original publication (Alkemade et al., 2022). All subsequent processing and analyses to extract laminar information, including ROI definition, data preprocessing, and layer delineation, were newly developed for this publication and constitute a novel, fully implemented pipeline that can serve as a template for future laminar investigations.

**Table 1.** Similarity of laminar profiles.

(LH)	Bielschowsky	Thionin	Parvalbumin
V1-V2	0.994	0.994	0.997
V1-V3	0,997	0.985	0.989
V2-V3	0.996	0.996	0.991
V1-hMT+	0.993	0.961	0.942
V2-hMT+	0.995	0.963	0.921
V3-hMT+	0.996	0.948	0.917
(RH)	Bielschowsky	Thionin	Parvalbumin
V1-V2	0.999	0.999	0.995
V1-V3	0.993	0.998	0.978
V2-V3	0.996	0.999	0.974
V1-hMT+	0.998	0.988	0.938
V2-hMT+	0.998	0.989	0.925
V3-hMT+	0.995	0.989	0.981

Correlations were computed to quantify the similarity of laminar profiles between each ROI (V1, V2, V3, and hMT+) for each microscopy contrast. Lower correlation values indicate greater dissimilarity between the laminar profiles of different regions. Significant differences were found between parvalbumin and Bielschowsky (*p* < 0.01), and between parvalbumin and Thionin (*p* < 0.01).

#### 2.1.2 In-vivo MRI dataset

The study was approved by the Ethics Review Committee of the Faculty of Psychology and Neuroscience (ERCPN) at Maastricht University, and all experimental procedures followed the principles of the Declaration of Helsinki. Written informed consent was obtained from our participant prior to the experiment. The in-vivo (f)MRI data were collected from the one participant (male, 25 years old) with the whole-body MAGNETOM 7T “Plus” (Siemens Healthineers, Erlangen, Germany) at Scannexus B.V. (Maastricht, The Netherlands) using a 32-channel RX head-coil (Nova Medical, Wilmington, MA, USA). The shimming procedure included the vendor-provided routines to maximize the field homogeneity within the imaging slab. This dataset consists of (1) whole-brain multi-echo $R_{2}^{*}$ anatomical images at 0.35 mm iso., (2) whole-brain MP2RAGE at 0.35 mm iso., and (3) single-slab covering visual areas resting-state fMRI data at 0.8 mm iso., collected over multiple sessions. Whole-brain multi-echo $R_{2}^{*}$ anatomical images were collected at 0.35 mm iso. resolution using a newly developed multi-echo multi-shot gradient recalled echo sequence with 3D echo planar imaging (EPI) readout (Gulban et al., 2026) (Fig. 1D). By combining the strength of a 3D acquisition and highly segmented k-space through a multi-shot technique, we could collect 0.35 mm iso. whole-brain anatomical images with very limited geometric distortions in less than 10 minutes acquisition time (6 minutes 48 seconds). The sequence multi-echo 3D EPI sequence (three echoes), hereby referred to as 3D ME EPI, consists of the following main parameters: field of view (FoV) = 200 x 200 x 130 mm; orientation = sagittal; bandwidth = 546 Hz/Px; repetition time (TR) as time between two excitation pulses = 52.8 ms; total acquisition time (TA) = 396 seconds; echo time (TE) = [9.76, 24.96, 40.16] ms; flip angle (FA) = 10°; Dual polarity = on; phase partial Fourier = off; Segmentation = 40; EPI factor = 5; PAT mode = CAIPIRINHA; Acceleration factor phase encoding (PE) x 3D = 3 x 2; CAIPI trajectory = w/o z-blips. The complete protocol is publicly available here: https://drive.google.com/file/d/1cV4qStubcjCCCxqXVFaZuwZemp4O1cqo/view?usp=drive_link. We collected a total of four runs. The MP2RAGE at 0.35 mm isotropic was collected in a separate session as “slab-stitched MP2RAGE” (Gulban, 2024). Briefly, five partial brain slabs at 0.35 mm iso. were concatenated to achieve whole-brain coverage. Each slab was collected within a single 10-minute run. The slabs were stitched in a post-processing step to have whole-brain images. In a separate session, resting-state fMRI data (rs-fMRI) were collected by using a 2D GE EPI sequence with blood oxygen level-dependent (BOLD) contrast (Moeller et al., 2010) with 0.8 mm isotropic resolution and coverage of the visual areas of interest (Fig. 1E). The in-plane field of view was 140 × 137 mm (176 × 172 matrix) for a total of 58 acquired slices. The imaging parameters were TE = 24.6 ms, TR = 2000 ms, flip angle FA = 69°, in-plane partial Fourier factor 6/8, GRAPPA = 3, multi-band (MB) = 2. A scanning protocol is available here: https://github.com/27-apizzuti/meso_MotionQuartet/blob/main/Protocols/sub-07_sess-01.pdf. We placed a small functional imaging slab according to a predetermined positioning based on results from a functional visual localizer obtained in an independent experimental session (Pizzuti et al., 2025) using the auto-align sequence (AAscout) from Siemens. Before the acquisition of the main run, we collected five volumes for distortion correction with the settings specified above but opposite phase encoding direction (posterior-anterior). A total of 300 volumes were collected in 10 minutes while the participant was asked to fixate a black fixation cross on a gray background. A frosted screen (distance from eye to screen: 99 cm; image width: 28 cm; image height: 17.5 cm) at the rear of the magnet was used to project the visual stimuli (fixation cross) (using Panasonic projector 28 PT-EZ570; Newark, NJ, USA; resolution 1920 x 1200; nominal refresh rate: 60 Hz) that participants can watch through a tilted mirror attached to the head coil.

### 2.2 Cortical mapping

Cortical mapping, including ROI definition and tissue segmentation, was performed using blockface images for post-mortem data and UNI images from MP2RAGE for in-vivo data, processed within BrainVoyager (Goebel, 2012). Specifically, we applied (1) the *Advanced Segmentation* and *Surface Reconstruction Pipeline* to segment the cortex and reconstruct the white matter surface, which allowed delineation of hMT+ using the Huang atlas (Huang et al., 2019), and (2) the *Cortex-Based Alignment Pipeline* for surface-based cortical alignment to the visfAtlas for defining V1, V2, and V3 (Rosenke et al., 2021). Tissue segmentation for both post-mortem and in-vivo data was refined through multiple iterations of manual corrections, performed independently by two researchers (A.P. and O.F.G.).

#### 2.2.1 Region-of-interest definition

Within BrainVoyager, the *Advanced Segmentation Pipeline* performs best on T1-weighted images at 0.5 mm isotropic resolution in ACPC space, optimal for subcortical masking. First, the contrast of the blockface images was inverted to resemble T1-weighted images. Then, within Brainvoyager, the blockface images were downsampled to 0.5 mm isotropic resolution (Sinc interpolation), and aligned to ACPC space for gray- and white-matter segmentation. Similarly, the UNI images were downsampled (Sinc interpolation) and aligned to ACPC space for segmentation. Once automatic segmentation was computed as result of the *Advanced Segmentation Pipeline*, manual refinement of the white matter segmentation was then performed by A.P. to remove mislabeled voxels that could create holes or false geometries, particularly around the occipital pole (e.g., near the sinus and subcortical areas). The resulting segmentation files, one from the blockface images and one from the UNI images, were subsequently used to reconstruct the white matter surfaces in BrainVoyager. On each surface, we defined hMT+ by manual drawing and by matching the characteristic macro-anatomical features reported by Huang et al. (e.g., cortical localization, cortical surface areas) (Fig. 2A, Supplementary Fig. S2). Note that hMT+ is only partially included in the visfatlas (one hemisphere was missing in the current release), this is why Huang atlas is chosen for hMT+ definition. Subsequently, each white matter surface (one post-mortem, one in-vivo) was used as input for the *Cortex-Based Alignment Pipeline* of BrainVoyager for aligning single-subject data to the visfatlas (Rosenke et al., 2021). By using visfatlas, we aimed to include the extent of the visual ROIs that can feasibly be stimulated during an fMRI, due to the reduced visual field that can be presented as stimulus in the scanner. Once the alignment to the visfatlas was successfully performed, the V1, V2, V3 ROIs were defined on each cortical white matter surface (Fig. 2A, Supplementary Fig. S2).

#### 2.2.2 Tissue segmentation

All ROIs (V1, V2, V3, hMT+) on cortical white matter surface were mapped back into the volume space (depth sampling -1 to +3 mm) within BrainVoyager and exported as NIFTI. For each dataset, ROIs and cortical tissues segmentation (white, gray matter and cerebro-spinal fluid) were projected back into the respective original native space (0.15 x 0.15 x 0.2 mm space for post-mortem dataset and 0.175 mm iso. for in-vivo dataset) using the program -greedy with “LABEL” interpolation option to preserve the binary nature of the data (Yushkevich et al., 2006). The final resolution for in-vivo data (0.175 mm iso.) was chosen to match the resolution of preprocessed $R_{2}^{*}$ map. Finally, a careful manual tissue segmentation around ROIs in the same native space was performed by A.P. and independently reviewed by O.F.G by using ITK-SNAP software (Yushkevich et al., 2006). Manual edits were lastly polished by using LN2_RIM_POLISH from LayNii (Huber et al., 2021) that implements a smoothing procedure using a combination of morphological operations of dilation and erosion. An exemplary slice showing the quality of our segmentation is reported in Figure 2B (ii). Within the final gray matter space, we projected the volume-based ROIs using LN2_VORONOI from LayNii. For the post-mortem microscopy data, a secondary segmentation was manually edited (by A.P. and independently reviewed by O.F.G) by refining the segmentation directly on each 2D microscopy slice at 0.2 mm iso. (later referred to as “2D segmentation”). While the initial segmentation refined using the blockface (later referred to as “3D segmentation”) provided a good overall alignment to individual slices, residual distortions from tissue stretching during sectioning hampered the accuracy of the 3D segmentation on 2D microscopy slices. Nevertheless, the 3D segmentation preserves tissue morphology and geometry and is well suited for extracting depth-specific information from qMRI. By contrast, the 2D segmentation corrects slice-specific misalignments and improves the accuracy of ROI and tissue boundaries at the microscopic level, albeit at the cost of distorting the global 3D morphology. For the aforementioned reasons, according to the specific analysis step presented in Section 2.3, one of the two segmentations is preferred.

### 2.3 Data preprocessing for laminar signal extraction

#### 2.3.1 Post-mortem dataset

We developed an ad-hoc analysis pipeline specifically for the publicly available post-mortem dataset (both microscopy and qMRI) from Alkemade et al. (2022) for addressing multimodal laminar questions. Specifically, the microscopy part required multiple stages of intensity normalization involving the development of three novel filters: (1) Tears Filter, (2) Local Bias Field Filter, and (3) Cutting Angle Filter to reduce artifacts due to slice cutting and compare the intensity of the three contrasts. Running these filters required a volumetric cortical parametrization with a geodesic 2D coordinate system encapsulating each ROI.

##### 2.3.1.1 Microscopy data

###### 2.3.1.1.1 Volumetric cortical parametrization

The tissue segmentation (both 3D and 2D segmentation) is also used to define a 3D geodesic coordinate system for each cortical region of interest. This parametrization is needed to run our novel geodesic filters (see Section *2.3.1.1.2*) for extracting laminar fine scale details in post-mortem microscopy data. We obtained the first two sets of geodesic coordinates (U,V coordinates) for the gray matter of each ROI separately, by running LN2_MULTILATERATE (input: 3D segmentation file). The third coordinate (coordinate D), parametrizing the cortical depth dimension, was computed by running LN2_LAYERS -equivol for each slice of each ROI (input: 2D segmentation file). While we ran the first program (LN2_MULTILATERATE) using the ROI segmentation computed on the 3D reconstructed model (see Section 2.2), the second program (LN2_LAYERS) was run iteratively for each 2D slice using the final segmentation refined for each slice. This choice is justified by the different implementations of the two programs: LN2_MULTILATERATE requires 3D information to compute the U and V coordinates, whereas LN2_LAYERS can use either 3D or 2D information to compute the D coordinate. For an extensive explanation on how the coordinates are computed, see Gulban et al. (2022). Although this approach for a volumetric parameterization of the cortex has been previously used in some recent layer-fMRI papers to investigate mesoscopic spatial features (Dresbach et al., 2024; Pizzuti et al., 2023, 2025), we extended it to microscopy data for the first time. For preserving the 2-dimensional nature of our data, we run our filters (Tears Filter, Local Bias Field Filter, Cutting Angle Filter) on each 2D slice separately by only using two instead of three coordinates, by setting one of the two (U, V) coordinates to a constant value (e.g., V = 1).

###### 2.3.1.1.2 Intensity normalization

**Fig. 3.**
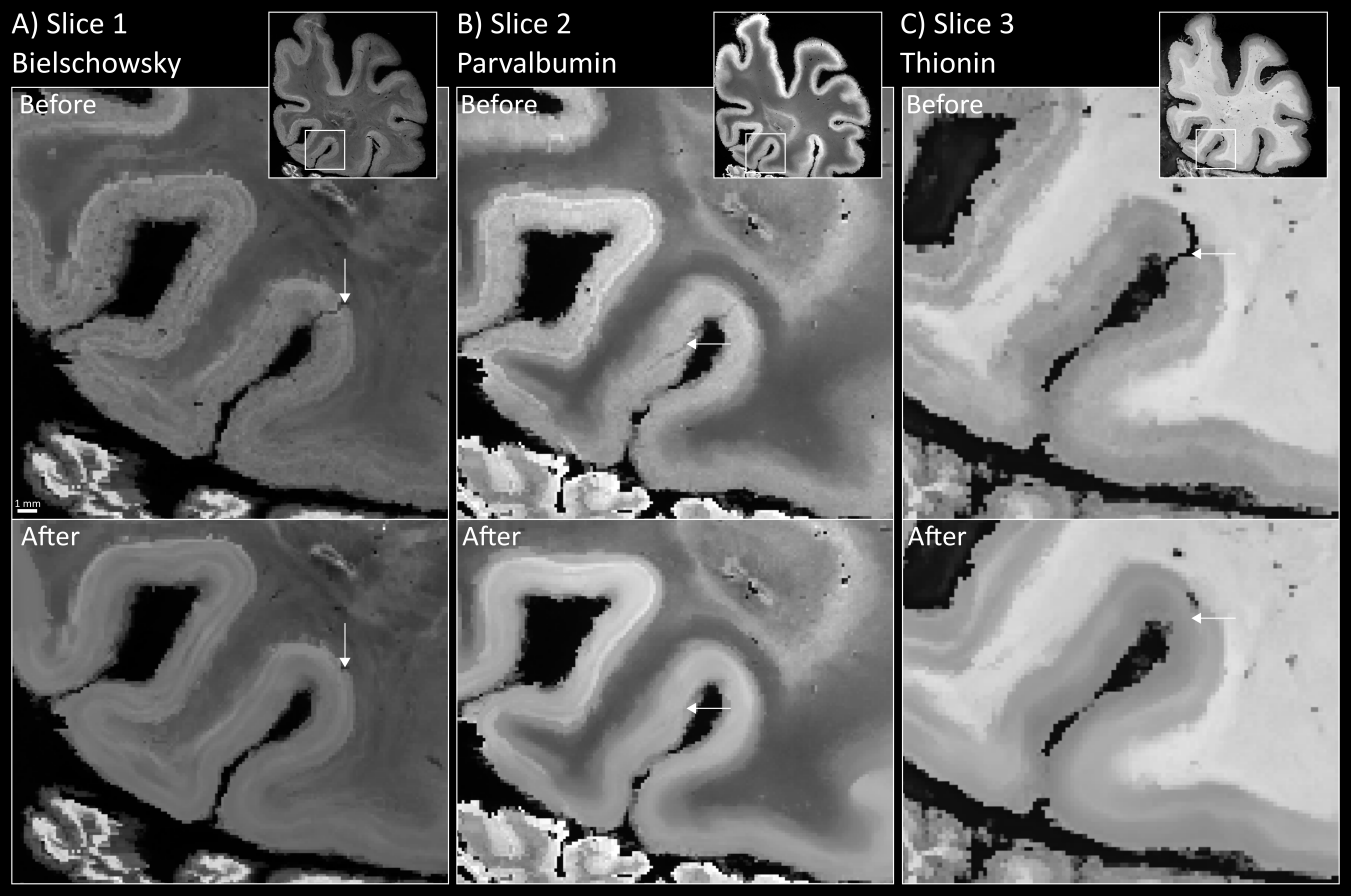
Tears filter. (A-B-C) Three exemplary slices spanning the three microscopy contrasts (Bielschowsky, parvalbumin, Thionin) showing before and after the application of the tears filter. White arrows pointed to the tears that we aimed to remove. Square inserts showed the entire slices from which we zoomed in to highlight the artifact.

**Fig. 4.**
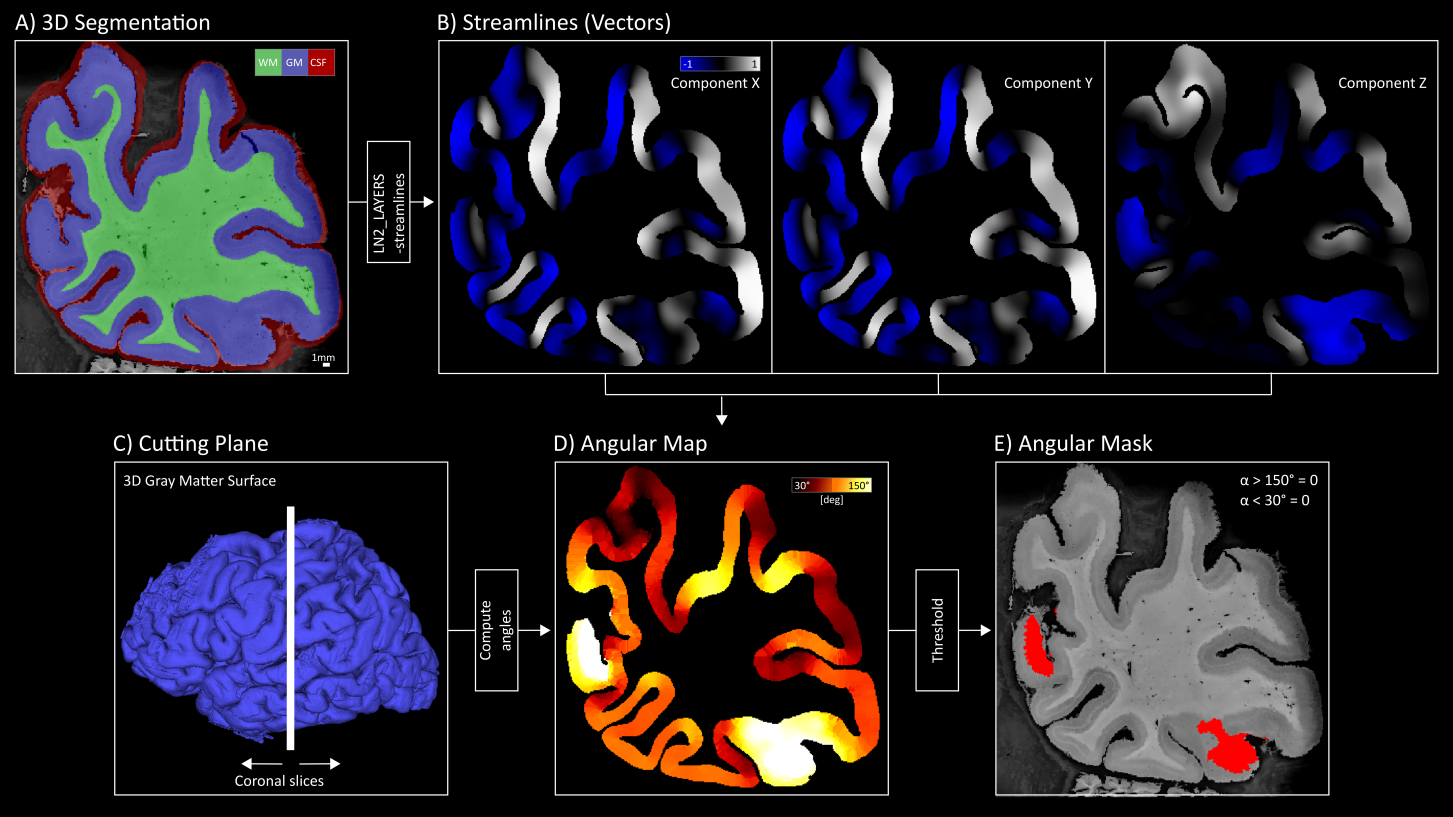
Cutting angle filter steps. (A) The 3D segmentation file is used as input for computing the streamlines using the program LN2_LAYERS with -streamlines flag. A tissue segmentation overlaid on a Nissl-stained slice is shown as an example. Colors indicate the three tissues, respectively: green for white matter, blue for gray matter, and red for cerebrospinal fluid. Note that in post-mortem data, the cerebrospinal fluid is physically not present and here indicates the outer boundary of gray matter. (B) Streamlines vector field. The three components (x, y, z) of the vector field indicate the local tissue orientation for each voxel is shown for the exemplary slice. (C) Schematic visualization of the cutting plane providing coronal slices as a vertical white line overlaid to the gray matter surface. (D) The angular map represents the output of the angular computation between streamlines (B) and cutting plane (C). (E) A threshold version of the map is shown as an angular mask; red patches indicate excluded areas for an angular measure exceeding 60° with respect to the cutting plane.

On the reconstructed 3D multi-contrast staining dataset, we applied three intensity normalization steps in order to enhance fine scale details and remove residual acquisition artifacts within the cortical landscape of each 2D microscopy slice. First, we performed a slice-by-slice intensity normalization based on percentile computation: for each voxel, we subtract the 5 percentile and divide by the difference between 95 and 5 percentile (Supplementary Fig. S1). In this way, the intensity range is uniform across slices and it is normalized between 0 and 1. Second, we applied the newly developed Tears Filter (Fig. 3) for removing high-frequency artifacts (e.g., tears, cracks). The Tears Filter is a geodesic low-pass filter with cylindrical kernel (radius: 0.5 and height: 10% of the local cortical depth measurement) implemented in LN2_UVD_FILTER -median within LayNii (Huber et al., 2021). Figure 3 shows three examples of how the filter mitigates the presence of the artifacts, which are irreversible distortions in the histology field that are induced by cutting, mounting, and staining (Fischl, 2013). Third, to mitigate the effect of cortical depth-dependent local bias leading with inhomogeneity affecting voxels spanning the same “geometrical cortical column”, we implemented a Local Bias Filter by estimating the local field bias around each voxel by using the same LayNii program (LN2_UVD_FILTER -median) but with a cylindrical kernel with a larger volume (radius: 0.5 and height: 100% of local cortical depth) and divided the voxel intensity by this estimated field. Sectioning post-mortem tissue can introduce misalignments when the angle between the cutting plane and the local cortical orientation deviates from perpendicular. Such deviations distort the laminar pattern, complicating comparisons across brain areas. To reliably extract cross-sectional information, sections should ideally be cut at 90^∘^ relative to the cortical surface—a principle already recognized by von Economo and Koskinas in the early 20th century, who dissected each gyrus and sulcus perpendicularly to its axis (Triarhou, 2013). Moreover, without this correction, misaligned sections can lead to overestimation of cortical thickness (Wagstyl et al., 2018). To address this issue, we developed a new filter, the Cutting Angle Filter, which automatically identifies and excludes gray matter regions that are poorly aligned with the coronal cutting plane (Fig. 4). Using the LN2_LAYERS -streamlines program from LayNii, we computed the local tissue orientation, generating a 4D vector map in which each voxel is assigned a radial vector connecting the inner and outer gray matter surfaces (locally orthogonal to the cortical surfaces). For this computation, we used 3D segmentation as it relies on 3D geometry of the brain (Fig. 4A). We then calculated a scalar angular map (Fig. 4D) by measuring the angle between each voxel’s local orientation (Fig. 4B) and the coronal cutting vector (Fig. 4C). Voxels exceeding an angle of 150° were excluded from subsequent laminar analysis (Fig. 4E), in line with Schleicher et al. (1999), who considered deviations of up to 60° from the vertical (90°) acceptable for preserving laminar integrity.

##### 2.3.1.2 qMRI data

For the qMRI part, the main artifact for the $R_{2}^{*}$ map within the post-mortem dataset was the presence of vessel residual artifacts that appear as very bright “bubbles” in the data. This is due to the air remaining trapped in the vessels when preparing a post-mortem sample for imaging (Fischl, 2013). We used an intensity-based histogram matching algorithm from ITK-SNAP segmentation tools to detect and exclude affected voxels from further analysis.

#### 2.3.2 In-vivo dataset

##### 2.3.2.1 Anatomical MRI

For the high-resolution 3D ME EPI anatomical images, we followed the established preprocessing pipeline from Gulban et al. (2026). First, to preserve fine scale details, we upsampled each run (and the three echo separately) to double resolution (0.175 mm iso.). We used the -upsample function from the greedy package with nearest-neighbor interpolation (Yushkevich et al., 2006). For each run separately, we computed the average of the three echos and used them as reference to bring the runs to the same space by using the linear and the non-linear registration program from greedy. The transformation matrix was then applied to each echo separately. Again, nearest neighbor was used as an interpolation step. Finally, we averaged the four runs and fitted a monoexponential decay function to compute the quantitative $R_{2}^{*}$ map. The anatomical images with UNI contrast at 0.35 mm iso. resolution, resulting from slab-stitching procedure, underwent the following processing steps: first, we applied a structure tensor denoising algorithm as implemented in the “Segmentator” python package (https://github.com/ofgulban/segmentator/wiki/Deriche-filter, Gulban et al., 2018) to increase the SNR (Gulban, 2024). Then, we upsampled to 0.175 mm iso. (as done for ME 3D EPI data) and we registered to the ME 3D EPI space using a non-linear co-registration procedure as implemented in greedy. Finally, we resampled our data using linear interpolation and used the resulting output as input for cortical mapping.

##### 2.3.2.2 Resting-state fMRI

Resting-state fMRI (rs-fMRI) data at 0.8 mm iso. underwent the following preprocessing steps: slice time correction (BrainVoyager), motion correction (BrainVoyager), distortion correction (FSL TOPUP), high-pass filter with three cycles (BrainVoyager). We averaged the time series and upsampled to 0.175 mm iso. to match anatomical resolution using the ndimage.zoom command from scipy (Virtanen et al., 2020) with spline interpolation (order 3). We coregister rs-fMRI data to high-resolution anatomical images (ME 3D EPI space) using a non-linear registration algorithm as implemented in greedy with linear interpolation. The temporal average of the fMRI time course is computed and used for laminar profiles. While this analysis discards the temporal information of the resting-state data, it does provide a measure of BOLD weighting.

### 2.4 Geometric cortical layers

The definition of cortical depth measures for both post-mortem and in-vivo dataset is based on the accurate tissue segmentation Figure 2B (ii) and was performed within LayNii using the LN2_LAYERS -equivol program. This program attributes a normalized (0–1) cortical depth measure to each voxel in 3D according to the equivolume principle (Fig. 2B (iii)). Only in the post-mortem data, we ran the algorithm iteratively on each 2D slice using the 2D segmentation (0.15 x 0.15 mm), since the 3D reconstruction inevitably comes with misalignments and geometrical deformation. We are aware that the equivolume principle is defined for 3D data; however, we qualitatively assessed that the number of errors arising by using a slice-by-slice approach was less compared by using the 3D reconstructed data. Note that most of the computational mistakes from the slice-by-slice layering procedure were automatically excluded from further analysis due to the Cutting Angle Filter. Laminar profiles were computed and shown as 2D histograms. The number of bins along the two dimensions (x-as cortical depth and y-as voxel intensity) was adjusted according to the region and the contrast represented. For each 2D histogram, we binned the cortical depth with 21 equivolume layers and overlaid a “median” laminar profile by computing the median intensity values across voxels belonging to each layer separately. Median was chosen over mean as it is more resilient to outliers that mostly affect tissue boundaries.

### 2.5 Statistical analysis on microscopy laminar profiles

**Fig. 5.**
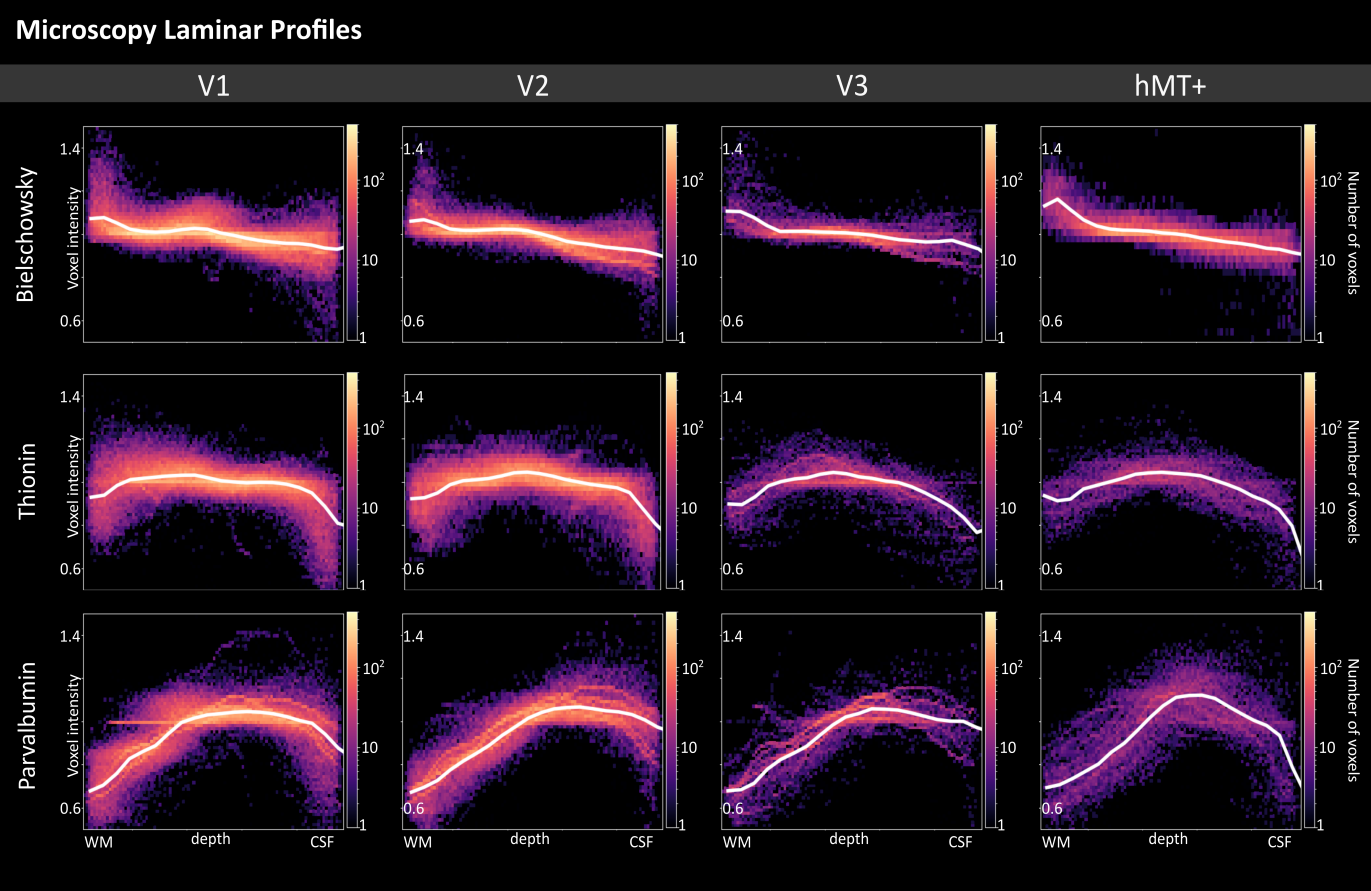
Laminar profiles for three microscopy contrasts (Bieloschowsky, Thionin, parvalbumin) are shown as 2D histograms for each ROI of the left hemisphere. Gray matter cortical depth measure is shown from white matter (x = 0) to cerebro-spinal fluid (x = 1) boundary from left to right. Solid white lines in each subplot show median intensity for 21 discrete equivolume layers. The Y-axis is shown within the 0.5–1.4 (a.u.) range for each subplot. Results from the right hemisphere are shown in Supplementary Figure S3.

We quantified the ability of each microscopy contrast (Bielschowsky, Thionin, parvalbumin) to differentiate visual areas. For each ROI (V1, V2, V3, hMT+) and stain contrast (Bieloschowsky, Thionin, parvalbumin), voxel-wise laminar profiles were first extracted (Fig. 5 and Supplementary Fig. S3) and then averaged, to obtain one representative profile per ROI and per stain type. For each stain and each pair of ROI-averaged profiles, a measure of correlation is computed (Table 1), providing a measure of similarity in laminar organization across regions given the same staining contrast. We performed a one-way ANOVA with stain type (Bielschowsky, Thionin, parvalbumin) as the grouping factor (columns of Table 1). The null hypothesis was that the three stains do not differ in their mean correlation coefficients between ROIs, that is, they are equally effective in discriminating visual areas. A significant ANOVA result, therefore, indicates that at least one stain differs in discriminability. To identify which stains differed, we conducted pairwise post-hoc comparisons using the Wilcoxon signed-rank test. A Bonferroni correction is applied for correcting for multiple comparisons (number of comparisons = 3).

### 2.6 Estimation of scaling factor between post-mortem and in-vivo ${\text{R}}_{\text{2}}^{*}$ laminar profiles

To evaluate the relative contributions of microstructure and vasculature to $R_{2}^{*}$, as the signal shifts from being influenced solely by microscopic factors (post-mortem) to a combination of microscopic and vascular contributions (in-vivo), we estimated a scaling factor between quantitative $R_{2}^{*}$ laminar profiles of the two datasets. This scaling factor was computed for each visual area as the ratio:

$$\text{scaling}=\frac{\beta_{\text{post}-\text{mortem}}}{\beta_{\text{in}-\text{vivo}}}$$ (1)

y(x)=β·x+ϵ (2)

**Fig. 6.**
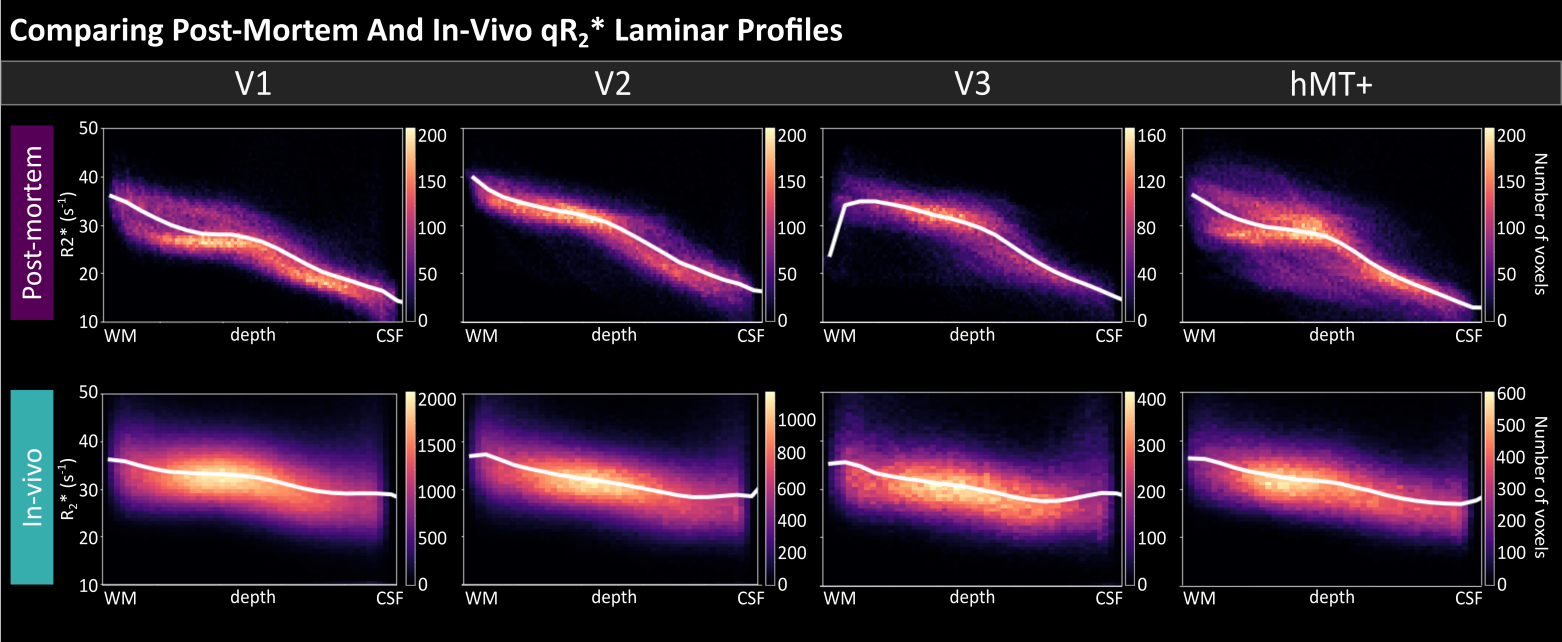
Post-mortem (top) and in-vivo (bottom) q $R_{2}^{*}$ laminar profiles are shown as 2D histograms for each ROI for the left hemisphere. Gray matter cortical depth measure is shown from white matter (x = 0) to cerebro-spinal fluid (x = 1) boundary from left to right. Solid white lines in each subplot show median intensity for 21 discrete equivolume layers. The Y-axis is shown within the 10–50 (s-1) range for each subplot. Results from the right hemisphere are shown in Supplementary Figure S4.

First, for each laminar profile (Fig. 6), we fit a simple linear regression model (Eq. 2) where *y* represents $R_{2}^{*}$ values (Table 2) as a function of cortical depth (indicated by the variable *x*), *β* is the slope, and ∈ is the residual error. This model assumes that the relationship between cortical depth and $R_{2}^{*}$ is linear and that the slope captures the laminar linear gradient of $R_{2}^{*}$. For each ROI, the slopes (*β*) were averaged across the two hemispheres and reported in Table 3. The ratio of the post-mortem to in-vivo slopes (Eq. 1) provided an estimate of the scaling factor between the $R_{2}^{*}$ laminar profiles of the two modalities. When examining post-mortem data, brain fixation must be considered. Although it substantially affects *R*_1_ values (Dinse et al., 2015), its impact on $R_{2}^{*}$ is found to be minimal (Deistung et al., 2016). The observed similar $R_{2}^{*}$ ranges suggest that differences between in-vivo and post-mortem $R_{2}^{*}$ mainly reflect variations in tissue composition.

**Table 2.** Mean quantitative $R_{2}^{*}$ [Hz] reported for three equivolume layers (deep, middle, superficial) for all the regions of interest in both post-mortem and in-vivo data.

	R2* [Hz] Post-mortem			R2* [Hz] In-vivo		
(LH)	Deep	Middle	Superficial	Deep	Middle	Superficial
hMT+	30.91	26.08	16.37	33.78	30.81	27.34
V1	30.83	26.25	18.12	33.97	32.11	29.14
V2	34.48	29.29	19.65	34.5	31.02	28.47
V3	33.87	29.21	18.94	32.96	29.74	28.05
(RH)	Deep	Middle	Superficial	Deep	Middle	Superficial
hMT+	32.62	27.11	17.27	34.23	31.3	26.93
V1	34.18	28.24	19.58	32.92	31.35	29.34
V2	37.82	31.56	20.41	34.38	31.18	28.55
V3	38.53	20.54	17.15	33.15	29.33	27.11

**Table 3.** Linear regression modeling for $R_{2}^{*}$ laminar profiles.

Post-mortem (slope)	V1	V2	V3	hMT+
Left hemisphere	-22.39	-24.87	-21.2	-24.74
Right hemisphere	-24.01	-28.51	-34	-26.33
Average	-23.2	-26.69	-27.6	-25.53
In vivo (slope)	V1	V2	V3	hMT+
Left hemisphere	-8.48	-8.91	-7.95	-10.01
Right hemisphere	-7.37	-10.11	-9.43	-11.16
Average	-7.92	-9.51	-8.9	-10.55
Ratio: Post-mortem/in vivo	3	2.8	3.1	2.4

The slope of the fitted curve is reported for each ROI. The ratio is considered as an estimate of the scaling factor between the two modalities.

## 3 Results

### 3.1 Comparing the microscopic cortical architecture of visual areas

We report the lamination patterns of four visual areas, V1, V2, V3, and hMT+, using three microscopy contrasts: Bielschowsky, Thionin, and parvalbumin (Fig. 5 and Supplementary Fig. S3). Bielschowsky staining correlates with fiber density and indirectly with myelin, while Thionin and parvalbumin provide cellular information by staining neuronal cell bodies and a subset of inhibitory interneurons, respectively. This analysis enables us to characterize each ROI by comparing the expression of these three microscopic features across cortical depths. We quantified the ability of each microscopy contrast to differentiate visual areas by calculating a measure of similarity (indexed as the pairwise correlation between each laminar profile; Table 1). Parvalbumin exhibited the lowest level of similarity across regions compared with Bielschowsky and Thionin, which was also qualitatively visible when comparing laminar profiles (Fig. 5 and Supplementary Fig. S3). To support our comparisons, we tested whether the correlations across the three groups are different: a one-way ANOVA shows significant differences across groups (*F* = 9.03, *p* = 0.0007). Pairwise comparisons using post-hoc Wilcoxon signed-rank tests revealed significant differences (after Bonferroni correction) between parvalbumin and Bielschowsky (*p* = 0.002) and parvalbumin and Thionin (*p* = 0.02), and no significant difference between Bielschowsky and Thionin (*p* = 0.1). Unlike cellular neuronal density, the distribution of parvalbumin neurons varies characteristically across cortical depths and ROIs. Although no direct functional inference can be drawn, the distribution of parvalbumin neurons across layers may be useful for interpreting layer-dependent fMRI signals, particularly in studies targeting inhibitory-related paradigms. In contrast, as differences in cortical myelination have historically been used to support cortical parcellation (e.g., Nieuwenhuys, 2013), one might, therefore, expect a myelin-sensitive contrast to provide strong differentiation between visual areas. However, because Bielschowsky staining does not specifically label myelin and instead reflects axonal density, regional differences primarily driven by myelin content may appear attenuated in this measure (Uchihara, 2007). This partial dissociation between fiber density and myelination has been demonstrated in demyelinating lesions in multiple sclerosis lesions, where Bielschowsky staining remains relatively preserved despite substantial myelin loss (Laule & Moore, 2018; Moore et al., 2000).

### 3.2 Comparing post-mortem and in-vivo quantitative $R_{\text{2}}^{*}$ laminar profiles

Bridging the gap between post-mortem and in-vivo studies is crucial for understanding the sources of $R_{2}^{*}$, as it is assumed that the signal shifts from being influenced solely by microscopic factors (post-mortem) to a combination of microscopic and vascular contributions (in-vivo). Therefore, in this study, we report the quantitative $R_{2}^{*}$ values across cortical depths for both post-mortem and in-vivo datasets (Fig. 6 and Supplementary Fig. S4). Table 2 accompanies Figure 6 and Supplementary Figure S4 by reporting the average $R_{2}^{*}$ value in only three equivolume layers. First, our $R_{2}^{*}$ cortical layer profiles are qualitatively consistent with previous reports in the visual cortex at the same spatial resolution, showing characteristic features such as a decrease toward the cortical surface and a peak around the middle layer (Gulban et al., 2022). Similar laminar $R_{2}^{*}$ profiles characterizing the visual cortex have also been observed across different resolutions in other studies (Kemper et al., 2018; Marques et al., 2017; McColgan et al., 2021), further supporting the robustness of these patterns. Second, we also observe qualitatively consistent profiles between the post-mortem and in-vivo data that show a consistent decrease of $R_{2}^{*}$ values from deep to the superficial layers. When comparing the magnitude of the $R_{2}^{*}$ laminar profiles between post-mortem and in-vivo data, we show that the post-mortem $R_{2}^{*}$ values are lower than those measured in vivo. In addition, the discrepancy between the $R_{2}^{*}$ values seem to be the highest in superficial layers. This discrepancy is expected, as in-vivo $R_{2}^{*}$ values include the additional contribution from blood vessels, leading to a faster signal decay. In contrast, post-mortem $R_{2}^{*}$ is influenced only by iron and myelin content, since the brain fixation process removes most of the blood, resulting in a slower decay. These differences are most pronounced from the middle to the superficial layers, which is expected due to the presence of more vasculature and partly by pial veins (partial volume effect). We estimate an average scaling factor across our visual ROIs between post-mortem and in vivo of 2.8 (Table 3). This means that the blood affects the estimation of $R_{2}^{*}$ with a factor of three: the slope of the $R_{2}^{*}$ laminar profiles in vivo is almost three times smaller than the one measured when microstructure only is considered (post-mortem).

### 3.3 Comparing resting-state fMRI and anatomical ${\text{T}}_{\text{2}}^{*}$ laminar profiles

**Fig. 7.**
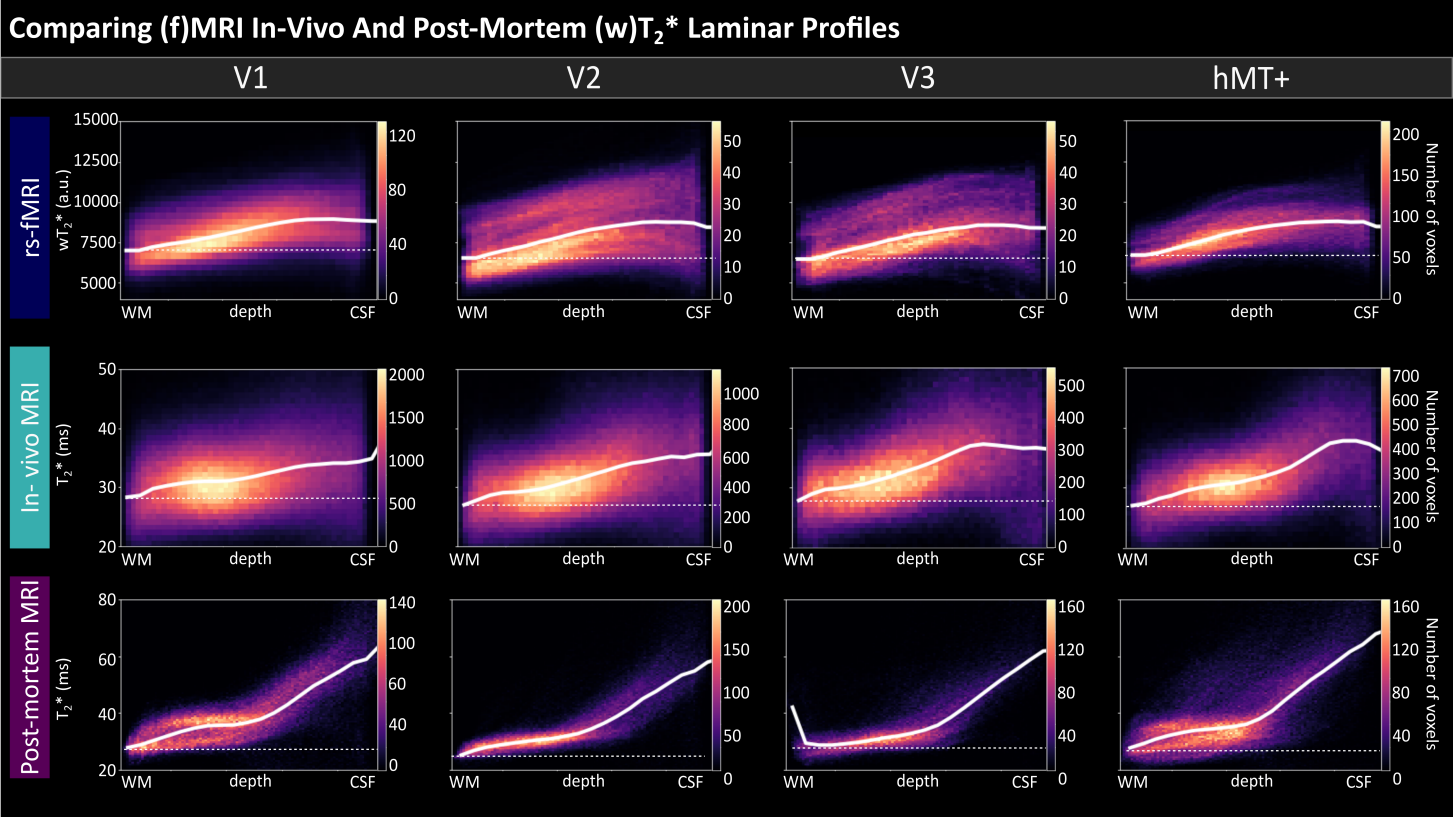
Resting-state fMRI as mean temporal signal (top), in-vivo $T_{2}^{*}$ (middle), and post-mortem $T_{2}^{*}$ (bottom) laminar profiles are shown as 2D histograms for each ROI. Gray matter cortical depth measure is shown from white matter (x=0) to cerebro-spinal fluid (x=1) boundary from left to right. Solid white lines in each subplot show median intensity for 21 discrete equivolume layers. Dotted lines indicate horizontal lines to highlight the increase toward superficial layers of main curves. The Y-axis range is displayed only in the first subplot of each data type and kept invariant across ROIs. Results from the right hemisphere are shown in Supplementary Figure S5.

Since GE-BOLD fMRI signals reflect variations of $T_{2}^{*}$ between conditions, we analyzed both anatomical and functional signal as $T_{2}^{*}$ variation (1/$R_{2}^{*}$). Here, our goal is to describe the laminar profiles extracted from the rs-fMRI run and to compare them with the anatomical $T_{2}^{*}$ laminar profiles from both in-vivo and post-mortem datasets (Alkemade et al., 2022) (Fig. 7 and Supplementary Fig. S5). Note that the data shown in the middle and bottom rows of Figure 7 are the same as those in Figure 6, but displayed as $T_{2}^{*}$ rather than $R_{2}^{*}$. In all ROIs, the temporal mean rs-fMRI signal exhibits a general increase toward the cortical surface, qualitatively resembling the anatomical profiles in both datasets. This result is consistent with previous reports (Guidi et al., 2020; Markuerkiaga et al., 2016, 2021; Pais-Roldán et al., 2020). Notably, the rs-fMRI data are acquired at lower spatial resolution (0.8 mm isotropic) compared with the anatomical $T_{2}^{*}$ (0.35 mm isotropic), which limits the ability to resolve finer laminar details, such as the characteristic dip around the middle layer observed in the anatomical profiles. Specifically, this characteristic dip in V1 observed with $T_{2}^{*}$ MRI is conventionally attributed to Stria of Gennari (Barbier et al., 2002; Duyn et al., 2007; Fukunaga et al., 2010; Gulban et al., 2022; Trampel et al., 2019). Accordingly, these profiles reflect broad laminar patterns, with future high-resolution fMRI offering the potential to resolve additional details (Feinberg et al., 2023; Vizioli et al., 2024). While our results suggest that the anatomical patterns observed in in-vivo and post-mortem data can also be detected in resting-state fMRI, it remains to be clarified whether such features consistently extend to task fMRI. We also acknowledge that in the present study, the combination of post-mortem $T_{2}^{*}$, in-vivo $T_{2}^{*}$, and BOLD data remains primarily qualitative; a more integrated analysis could provide stronger mechanistic insight into how vascular and microstructural contributions jointly shape laminar profiles for functional outcome. However, methodological differences in acquisition resolution and contrasts currently limit direct quantitative integration, and addressing this opportunity will require multimodal datasets at matching resolution, which we see as a promising direction for future work.

## 4 Discussion

### 4.1 Summary

In this study, we propose a multimodal laminar characterization for four human visual areas (V1, V2, V3, hMT+) that aims to bridge the micro and mesoscale. We used the novel publicly available post-mortem dataset, which uniquely presents multiple microscopy contrasts and quantitative MRI for the same individual, and complement it with our in-vivo dataset consisting of both high-resolution anatomical and functional MRI (Fig. 1). We investigated the microscopic underpinning of our regions of interests by analyzing the cortical variation of three microscopy contrasts (Bielschowsky, Thionin, parvalbumin) and found a central role for parvalbumin for area differentiation (Fig. 5, Supplementary Fig. S3). Moreover, we compared $R_{2}^{*}$ MRI cortical variation of post-mortem with in-vivo samples, and found a common linear decrease $R_{2}^{*}$ across the two modalities (Fig. 6, Supplementary Fig. S4). Finally, we report the laminar profiles in rs-fMRI (Fig. 7, Supplementary Fig. S5) and compare it with the structural laminar profiles measured in the same living brain and in post-mortem brain. Although at different spatial resolution, the same overall characteristic positive slope characterized the three laminar profiles.

### 4.2 Implications for layer-fMRI

#### 4.2.1 Cortical distribution of parvalbumin interneurons

It is generally assumed that the neural activity of excitatory neurons is reflected in BOLD fMRI response, since excitatory neurons constitute 80–90% of all cortical neurons (Meyer et al., 2011). However, it is known that the ratio between excitatory and inhibitory neurons varies across cortical depth and areas (Markram et al., 2004; Tremblay et al., 2016). Even though our microscopy data cannot quantify the ratio of excitatory and inhibitory neurons, we still found it notable that the cortical distribution of parvalbumin neurons is a characteristic feature of the region of interest (Fig. 5). Parvalbumin neurons, even if they represent only one category of interneurons, they are the most abundant GABAergic neurons in the cortex (Rudy et al., 2011). Moreover, parvalbumin neurons are assumed to have indirect influence through the inhibition of pyramidal cells which is responsible of vasoconstriction (Lee et al., 2021). Due to this property, neural mechanisms involving this category of interneurons can affect the hemodynamic response detected by fMRI (Moon et al., 2021), with optogenetic stimulated PV neurons eliciting a biphasic hemodynamic response (Vo et al., 2023). While the possibility of resolving the functional contributions of specific neuronal populations using submillimeter fMRI seems feasible based on invasive animal studies, in humans this approach is still in its very early stages (Kim, 2025). In this context, developing an anatomical map of different neuronal types (e.g., the distribution of parvalbumin interneurons) across cortical layers and areas could provide a crucial framework to guide functional investigations using layer-specific fMRI signals. For instance, Torres-Gomez et al. (2020) suggested that changes in the proportion of parvalbumin neurons in layers 2/3 may support the emergence of activity encoding working memory in association areas in primate brains. We, therefore, anticipate that integrating proxies for microstructural neuronal information in humans will enhance the interpretation of laminar functional responses during the execution of complex tasks.

#### 4.2.2 Vascular vs microstructural gray matter composition

When reaching submillimeter spatial resolution in fMRI, signals from different cortical laminae can be disentangled. However, at this spatial scale also other mesoscopic details regarding the underlying vascular network have to be considered while discussing layer-fMRI results. In particular, the BOLD signal reflects variation of $T_{2}^{*}$ mainly coming from veins (Koopmans & Yacoub, 2019; Uludag & Havlicek, 2021). The presence of pial veins together with intracortical veins usually induces a bias known as “draining vein” effect that is manifested as a signal that linearly increases towards the cortical surface. This is due to the fact that the blood is drained from deep to superficial layers by the veins that accumulate signals while traveling upward, making the identification of the neural laminar source challenging (Koopmans & Yacoub, 2019). This cortical trend has been reported for both task-induced (Aitken et al., 2020; Fracasso et al., 2018; Mourik et al., 2021) and resting-state activity (Guidi et al., 2020; Markuerkiaga et al., 2021; Pais-Roldán et al., 2020). In this study, we showed that a linear trend characterizes the gray matter structure both in-vivo and post-mortem q $T_{2}^{*}$ laminar profiles (Fig. 7). When the vasculature is taken out of the equation, as in the post-mortem case, $T_{2}^{*}$ is still the highest at superficial layers. This result suggests that microstructural properties (e.g., myelin, iron) contribute to the linear increase in addition to vasculature in both task and resting-state fMRI, a component currently not accounted for in generative models of layer-fMRI (Havlicek & Uludağ, 2020; Markuerkiaga et al., 2016), which still assume constant extravascular $T_{2}^{*}$ across depths (see Section 4.2.3 and Appendix). As layer-fMRI progresses to higher resolution (< 0.5 mm isotropic) (Feinberg et al., 2023; Vizioli et al., 2024), disentangling vascular from microstructural contributions will be essential to refine such models and improve mechanistic interpretations. Given that regional and local differences in the relative weighting of vascular and microstructural contributions are likely, understanding how these elements consistently or differentially shape $T_{2}^{*}$ profiles across cortical regions will be key to advancing laminar fMRI. A scenario where both factors together produce a consistent linear increase across the cortex would align with prior observations, while also underscoring the importance of further investigation. Extending generative laminar models to embed area-specific microstructural as well as vascular features (such as $T_{2}^{*}$ laminar profiles) will, therefore, be crucial for improving our understanding of the sources of variance driving layer-fMRI.

#### 4.2.3 Consequences for models of layer-fMRI

Generative laminar models are useful tools to predict layer-fMRI dynamics. However, the complexity and the accuracy of those models depend on the assumptions and on the physical parameters estimates (Havlicek & Uludağ, 2020; Markuerkiaga et al., 2016; Uludag et al., 2009). Below, we discuss our resting-state laminar profiles together with the generative laminar model from Havlicek and Uludağ (2020). The model uses two compartments (intravascular (blood) and extravascular (parenchyma)) to describe the BOLD signal generated by a GE sequence (Eq. A1, Appendix). T1 effects are neglected in this model. As the signal from the intravascular compartment can be neglected at 7T (due to $R_{2}^{*}$), the predicted laminar profile depends only on the extravascular compartment. According to this modeling, the variables affecting the signal are:

1. S0 or rho, baseline signal intensity measured at TE=0 ms, when no relaxation (particularly $T_{2}^{*}$ relaxation) has taken place. This signal measures tissue water proton density and includes contributions from transmit efficiency and receive sensitivity biases.
2. CBV, cerebral blood volume, measuring the fraction of cerebral blood volume within a given amount of brain tissue.
3. $R_{2}^{*}$, the transverse relaxation rate, which reflects how quickly the MRI signal decays in a gradient-echo sequence.

In laminar BOLD signal models, it is often assumed for simplicity that S0 and $R_{2}^{*}$ remain constant across cortical depth, while cerebral blood volume (CBV) increases from deep to superficial layers (see Table 2 from Havlicek & Uludağ, 2020). Under this set of assumptions, the model predicts a signal profile that decreases from deep to superficial layers (see Appendix – Laminar resting-state fMRI). However, the empirical laminar resting-state fMRI profile observed in our data shows the opposite trend (Fig. 7, Supplementary Fig. S5). Similar depth-dependent signal profiles have also been reported in previous studies (Guidi et al., 2020; Markuerkiaga et al., 2016, 2021; Pais-Roldán et al., 2020). This discordance between modeling and empirical data points to the obvious conclusion that some of the above assumptions have to be relaxed in order to counterbalance the effect of CBV. In this work, we focus on quantitative $R_{2}^{*}$ and consistently show in both post-mortem and in-vivo $R_{2}^{*}$ is clearly modulated (and not constant) with respect to cortical depth. The laminar variation of $R_{2}^{*}$ with its characteristic decrease from deep to superficial layers is one factor that counterbalances the effect of CBV: as $R_{2}^{*}$ decreases with cortical depth, the $T_{2}^{*}$-weighted rs-fMRI signal increases with cortical depth. Together with $R_{2}^{*}$, it is plausible to think that S0 also changes across cortical depth as a concurrent contribution to the resulting laminar profile measured during a rest condition. Empirical data, such as our observed laminar profiles, are invaluable in challenging and refining these models, offering insights that purely theoretical approaches may overlook. Notably, the range of $R_{2}^{*}$ variation we observe at rest (3–$6{\text{s}}^{-1}$ ) is comparable to task-related laminar $\Delta R_{2}^{*}$ reported in the literature (e.g., 0.5–$3{\text{s}}^{-1}$ in V1; Markuerkiaga et al., 2021), indicating that resting-state laminar $R_{2}^{*}$ differences represent a meaningful source of variance that modeling must account for. While we cannot yet quantify the exact impact on task-evoked percent signal changes, these baseline differences are clearly non-negligible. A full set of modeling simulations is necessary to systematically explore the combined effects of $R_{2}^{*}$ and S0 on laminar profiles, which could help resolve the divergence between current model predictions and empirical findings. Adjusting these parameters in future work may allow models to more accurately capture the interactions that shape laminar BOLD signals across cortical depth, improving predictions of layer-fMRI dynamics.

### 4.3 Extending open access tools for the post-mortem dataset

Publicly available post-mortem datasets are invaluable for studying the microscopic structure of the human brain (Alkemade et al., 2022; Amunts et al., 2013). However, developing specialized analysis toolboxes is also essential for correctly extracting information from these rare datasets and integrating it with other modalities. For instance, the Allen Brain Map portal (https://portal.brain-map.org/overview) is complemented by a suite of publicly available analysis tools (https://github.com/AllenInstitute). Following the same rationale, our streamlined analysis pipeline released as an open github repository (https://github.com/27-apizzuti/multimodal_layers.git) extends the open access analysis tools for the post-mortem dataset provided by Alkemade et al., offering new tools for extracting laminar information. To enhance the laminar details and ensure robust cortical sampling from multiple slices, we developed the “cortical tears filter” (Fig. 3) and the “cutting angle filter” (Fig. 4). These tools are crucial for deriving reliable contrast- and ROI-specific laminar information. While we acknowledge the existence of other software addressing similar artifacts (Kindle et al., 2011; Mancini et al., 2020; Schleicher et al., 1999), we highlight that our implementation is designed to encourage widespread use of this post-mortem dataset specifically by offering tools that can be applied on the downloaded data. Note that the tissue segmentation step is not fully automated and requires expertise and manual work. This is a crucial step for accurate and precise layer profiles when working with very high-resolution images. With all analysis methods and data used in this paper (histology, qMRI, and layer-fMRI) being publicly accessible, our framework offers a valuable resource for researchers conducting similar studies in other brain regions. By facilitating the study of cortical laminar structure and integrating this with functional information, our approach supports a deeper understanding of the structure–function relationships across the cortex, potentially uncovering unique regional patterns that contribute to brain function and disease.

### 4.4 Limitations and conclusions

Although our efforts to bridge scales and techniques have led to new insights and discussions on the laminar organization of four visual areas, it is worth pointing out the limitations of our results. Firstly, we focused on a limited subset of visual regions (V1, V2, V3, hMT+), which restricts the scope of our findings from drawing widespread conclusions on laminar features and visual hierarchy. Secondly, we defined the ROIs for V1, V2, and V3 using the visfatlas (Rosenke et al., 2021) with cortex-based alignment, and hMT+ was delineated based on macro-anatomical criteria outlined in Huang et al. (2019). While we acknowledge that different methodologies can lead to partially overlapping regional boundaries (Turner, 2013a, 2019), we argue that these discrepancies are unlikely to impact our results as we aim to understand regional and not local lamination differences. Additionally, comparing post-mortem and in-vivo tissue introduces limitations due to the effects of brain extraction and fixation. These effects could influence the $R_{2}^{*}$ measurements (Deistung et al., 2016) and may alter the apparent thickness of cortical layers due to slight tissue shrinkage during fixation (Mouritzen Dam, 1979). Moreover, as only two human brains were analyzed to compare post-mortem and in-vivo tissue features such as laminar variations of $T_{2}^{*}$, it remains unclear how much of the observed variability reflects inter-individual differences, given, for instance, the age mismatch between the post-mortem brain (59 years) and the in-vivo brain (25 years). Consequently, having two brains limits the generalizability of our findings, as individual anatomical variations may not be fully represented. We note that such small sample sizes are common in high-resolution post-mortem studies due to current technical constraints (e.g., the BigBrain dataset, Amunts et al., 2013, is based on a single brain). While small samples are clearly insufficient for generalization, these datasets offer a rare opportunity to explore laminar patterns that are otherwise difficult to obtain. Future studies with larger sample sizes will be necessary to evaluate inter-individual variability and further validate the generalizability of these findings. By providing an open-access analysis pipeline for the both the post-mortem dataset (Alkemade et al., 2022) and our in-vivo dataset, we offer a novel framework for extracting laminar information and integrating it with other modalities, facilitating future studies of cortical structure–function relationships. Future research could focus on expanding the multimodal characterization to include additional visual areas and functional contexts, enhancing our understanding of the dynamic interplay between micro- and mesoscale features in visual processing.

## Supplementary Material

Supplementary Material

## Data Availability

Analysis code is available on GitHub: https://github.com/27-apizzuti/multimodal_layers.git. Refer to Alkemade et al. (2022) for raw post-mortem data. In-vivo anatomical data are shared in Zenodo: https://doi.org/10.5281/zenodo.14147820. Raw resting-state laminar-fMRI data are shared in Zenodo: https://doi.org/10.5281/zenodo.14164885. Preprocessed data from both post-mortem and in-vivo datasets are shared in Zenodo: https://doi.org/10.5281/zenodo.14164885

## Appendix

The laminar BOLD signal equation (Eq. 5 from Havlicek & Uludağ, 2020) for a cortical layer (denoted by subscript *k*) for a baseline condition (denoted by subscript *baseline*; note that in the original Havlicek & Uludağ, 2020 paper the subscript for indicating baseline is “0”) is given as

$$S_{baseline}*=\left(1-\underset{i}{\sum \;}V_{baseline,i,k}\right)S_{baseline,Ek}*+\underset{i}{\sum \;}V_{baseline,i,k}S_{baseline,Ik}$$
(A1)

where

1. $V_{baseline,i,k}$ is the cerebral blood volume from the vascular compartment (denoted by subscript *i*) at baseline $CB{\text{V}}_{baseline,i,k}$ for the *k*-layer.
2. The vascular compartment includes the venules and ascending veins compartments.
3. *S*_*baseline*, *I k*_ is the intravascular signal at baseline for the *k*-layer.

### Laminar resting-state fMRI

Under the approximation that, at 7T, $R_{2}^{*}$ of the blood (intravascular) is twice as fast as $R_{2}^{*}$ of the parenchyma, we can assume that $S_{\text{rest},Ik}\text{​}\;=0$ (Havlicek & Uludağ, 2020).

Thus, we can write

$$S_{\text{rest},k}=\left(1-\sum V_{rest,i,k}\right)S_{\text{rest},Ek}$$
(A2)

Since the parenchyma $R_{2}^{*}$ (extravascular) is assumed to be constant across cortical depth, we approximate $S_{\text{rest},Ek}$ to be constant as well across cortical depth (Havlicek & Uludağ, 2020).

$$S_{\text{rest},k}\approx \left(1-\sum_{i}\;V_{rest,i,k}\right)$$
(A3)

As the cerebral blood volume is expected to linearly increase across cortical depth (Havlicek & Uludağ, 2020), the resulting signal at resting state is expected to decrease as a function of cortical depth.
