# Supplementary material for: Multimodal laminar characterization of visual areas along the cortical hierarchy
Source: Imaging Neurosci (Camb). 2026 Jul 14;4:IMAG.a.1279. doi: 10.1162/IMAG.a.1279 (PMC13370752; doi:10.1162/IMAG.a.1279)
Supplement: Supplementary Material [file IMAG.a.1279_supp.pdf]

Supplementary Material

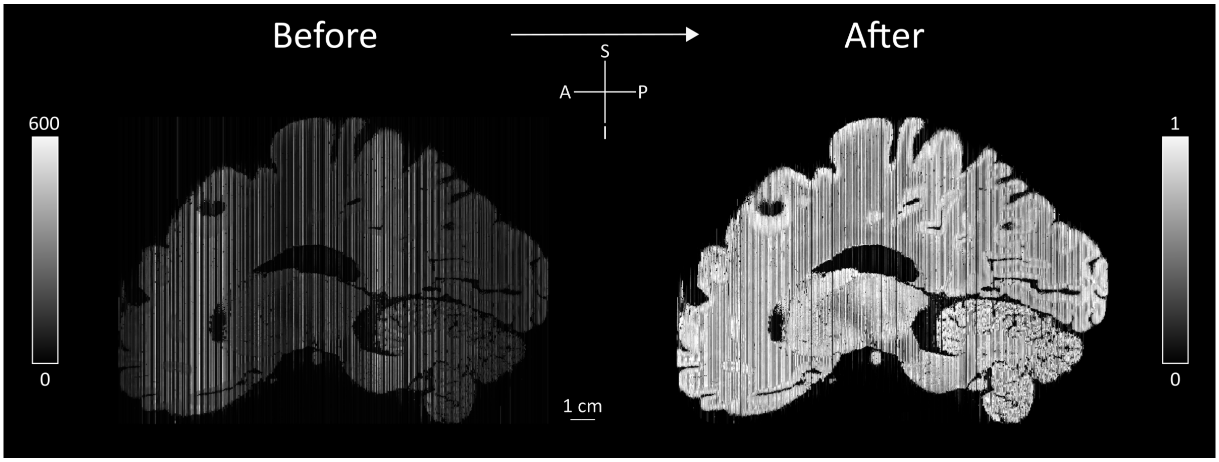

Supplementary Figure 1: Slice-based percentile intensity normalization. Comparing post-mortem stack microscopy data (sagittal view) before and after this step. The intensity of each slice is normalized between 0-1.

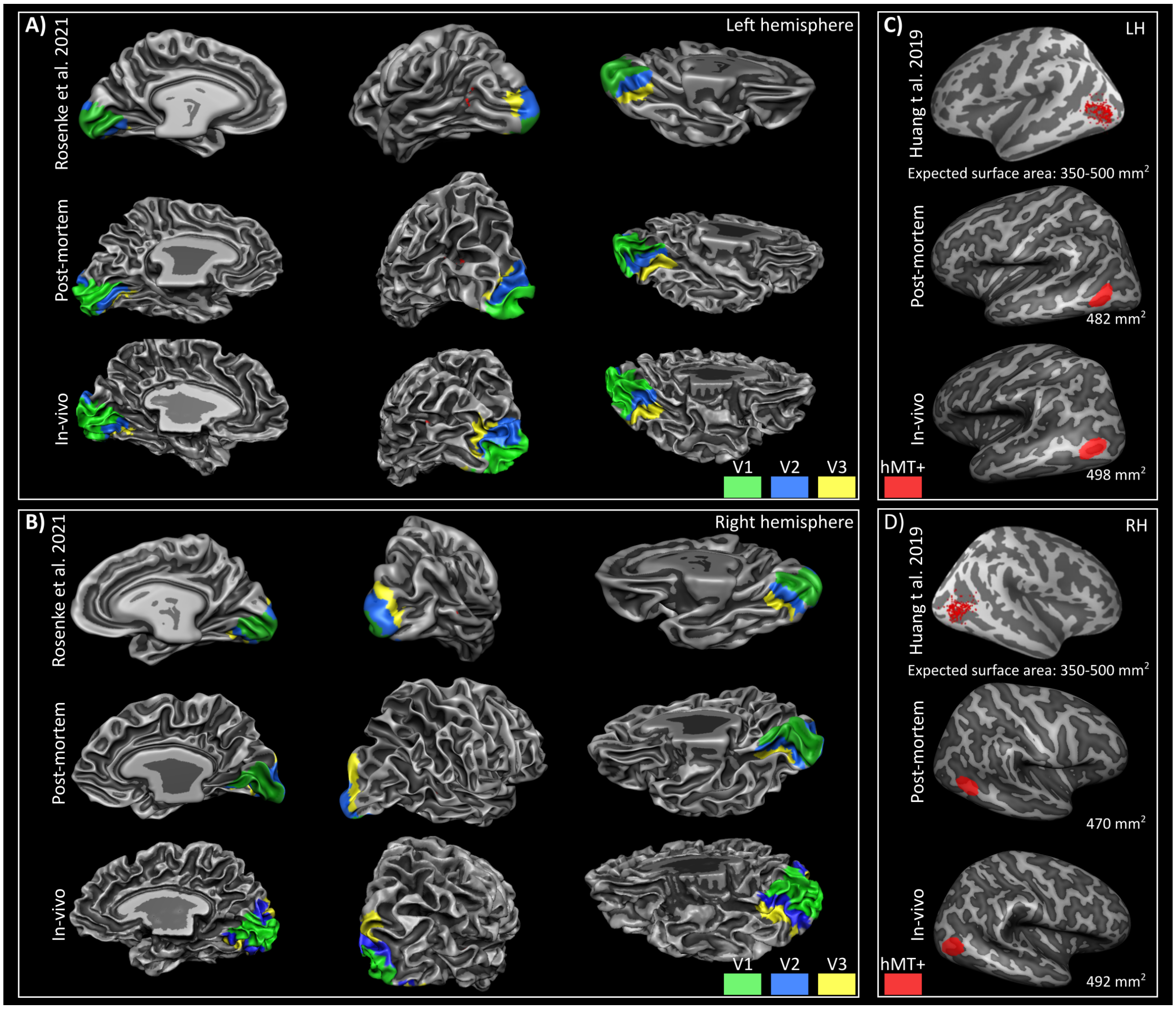

Supplementary Figure 2: Extended ROI definition for both post-mortem and in-vivo datasets. Results from cortex-based alignment to the visual functional atlas (**Rosenke**) are shown on a white matter surface for both the left (A) and the right (B) hemisphere. Results from the macro-anatomical definition of hMT+ as explained in Huang et al. (2019) are shown on the inflated white matter surface for both the left (C) and the right (D) hemisphere. Surface area is reported for each hMT+ ROIs.

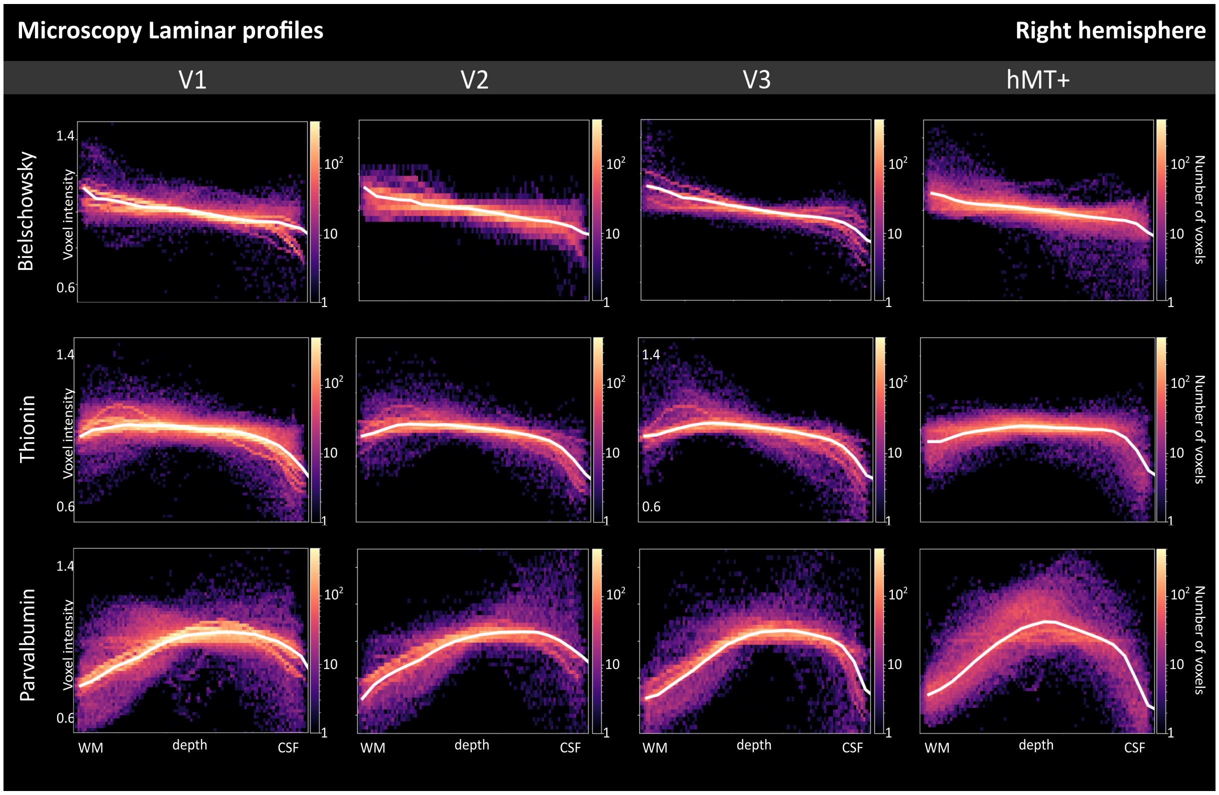

Supplementary Figure 3: Laminar profiles for three microscopy contrasts (Bieloschowsky, Thionin, parvalbumin) are shown as 2D histograms for each ROI of the right hemisphere. Gray matter cortical depth measure is shown from white matter (x = 0) to cerebro-spinal fluid (x = 1) boundary from left to right. Solid white lines in each subplot show median intensity for 21 discrete equivolume layers. The Y-axis is shown within the 0.5-1.4 (a.u.) range for each subplot. Results from the left hemisphere are shown in main **Figure 5**.

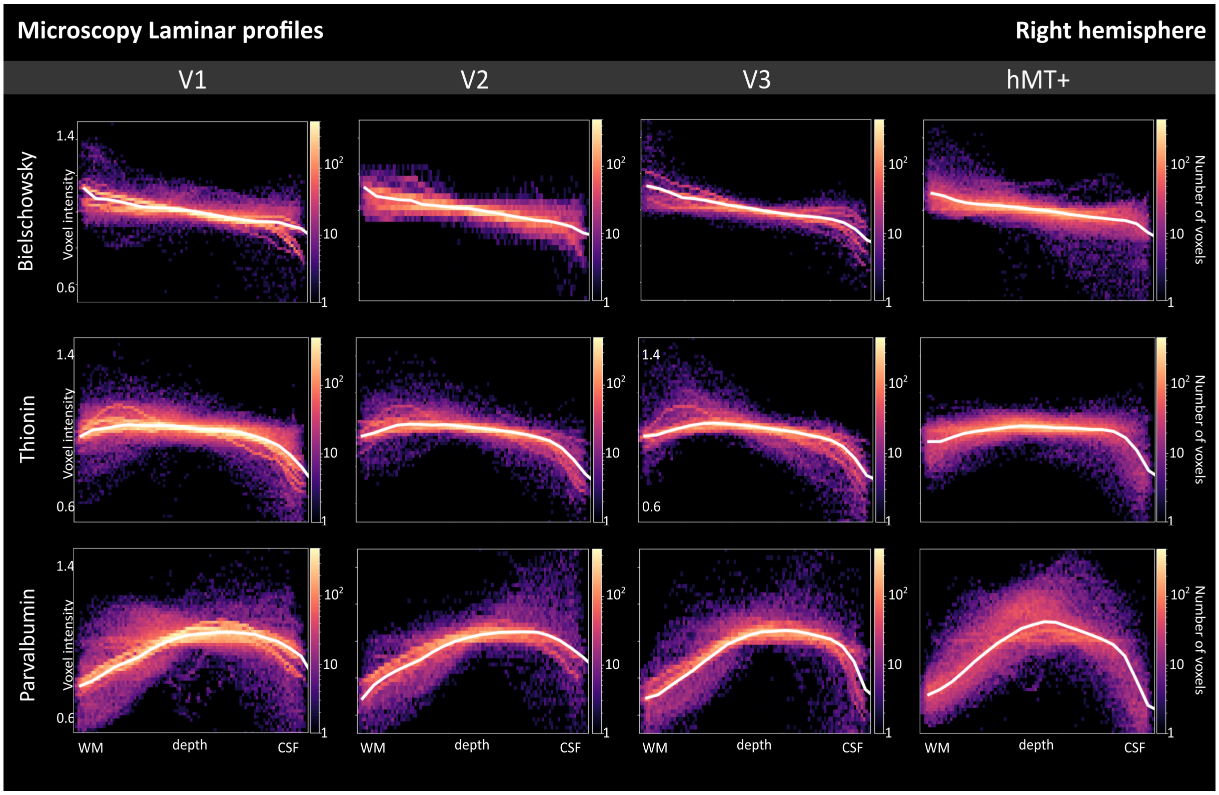

Supplementary Figure 4: Post-mortem (top) and in-vivo (bottom) qR_2^* laminar profiles are shown as 2D histograms for each ROI for the right hemisphere. Gray matter cortical depth measure is shown from white matter (x = 0) to cerebro-spinal fluid (x = 1) boundary from left to right. Solid white lines in each subplot show median intensity for 21 discrete equivolume layers. The Y-axis is shown within the 10-50 (s-1) range for each subplot. Results from the left hemisphere are shown in main **Figure 6**.

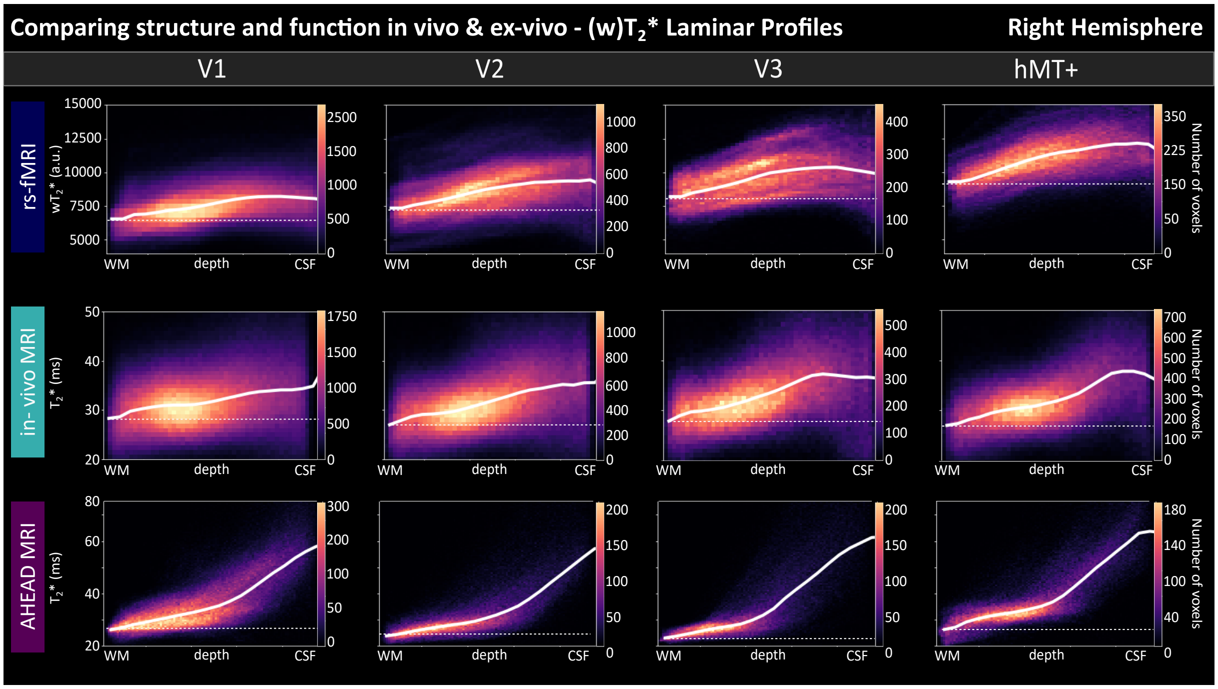

Supplementary Figure 5: Resting-state fMRI as mean temporal signal (top), in-vivo T_2^* (middle) and post-mortem T_2^* (bottom) laminar profiles are shown as 2D histograms for each ROI for the right hemisphere. Gray matter cortical depth measure is shown from white matter (x = 0) to cerebro-spinal fluid (x = 1) boundary from left to right. Solid white lines in each subplot show median intensity for 21 discrete equivolume layers. Dotted lines indicate horizontal lines to highlight the increase towards superficial layers of main curves. The Y-axis range is displayed only in the first subplot of each data type and kept invariant across ROIs. Results from the left hemisphere are shown in main **Figure 7**.
